# Validation of Morphometric Analyses of Small-Intestinal Biopsy Readouts in Celiac Disease

**DOI:** 10.1371/journal.pone.0076163

**Published:** 2013-10-11

**Authors:** Juha Taavela, Outi Koskinen, Heini Huhtala, Marja-Leena Lähdeaho, Alina Popp, Kaija Laurila, Pekka Collin, Katri Kaukinen, Kalle Kurppa, Markku Mäki

**Affiliations:** 1 Tampere Center for Child Health Research, University of Tampere and Tampere University Hospital, Tampere, Finland; 2 Tampere School of Health Sciences, University of Tampere, Tampere, Finland; 3 Institute for Mother and Child Care “Alfred Rusescu” and University of Medicine and Pharmacy “Carol Davila”, Bucharest, Romania; 4 Department of Gastroenterology and Alimentary Tract Surgery, Tampere University Hospital, Tampere, Finland; 5 Department of Medicine, Seinäjoki Central Hospital, Seinäjoki; 6 School of Medicine, University of Tampere, Tampere, Finland; Tulane University, United States of America

## Abstract

**Background:**

Assessment of the gluten-induced small-intestinal mucosal injury remains the cornerstone of celiac disease diagnosis. Usually the injury is evaluated using grouped classifications (e.g. Marsh groups), but this is often too imprecise and ignores minor but significant changes in the mucosa. Consequently, there is a need for validated continuous variables in everyday practice and in academic and pharmacological research.

**Methods:**

We studied the performance of our standard operating procedure (SOP) on 93 selected biopsy specimens from adult celiac disease patients and non-celiac disease controls. The specimens, which comprised different grades of gluten-induced mucosal injury, were evaluated by morphometric measurements. Specimens with tangential cutting resulting from poorly oriented biopsies were included. Two accredited evaluators performed the measurements in blinded fashion. The intraobserver and interobserver variations for villus height and crypt depth ratio (VH:CrD) and densities of intraepithelial lymphocytes (IELs) were analyzed by the Bland-Altman method and intraclass correlation.

**Results:**

Unevaluable biopsies according to our SOP were correctly identified. The intraobserver analysis of VH:CrD showed a mean difference of 0.087 with limits of agreement from −0.398 to 0.224; the standard deviation (SD) was 0.159. The mean difference in interobserver analysis was 0.070, limits of agreement −0.516 to 0.375, and SD 0.227. The intraclass correlation coefficient in intraobserver variation was 0.983 and that in interobserver variation 0.978. CD3^+^ IEL density countings in the paraffin-embedded and frozen biopsies showed SDs of 17.1% and 16.5%; the intraclass correlation coefficients were 0.961 and 0.956, respectively.

**Conclusions:**

Using our SOP, quantitative, reliable and reproducible morphometric results can be obtained on duodenal biopsy specimens with different grades of gluten-induced injury. Clinically significant changes were defined according to the error margins (2SD) of the analyses in VH:CrD as 0.4 and in CD3^+^-stained IELs as 30%.

## Introduction

In celiac disease the characteristic gluten-induced small-intestinal mucosal injury develops gradually [1], [2]. The spectrum of mucosal changes contains two separate measurable parameters, inflammation reflected by intraepithelial lymphocytic infiltration and morphological damage which includes villous atrophy and crypt hyperplasia. The mucosal lesion is the gold standard for diagnosing celiac disease and is present in patients both with and without clinical symptoms or signs of the disease. Well-known grouped classifications in histological assessment are one described by Marsh and modified by Oberhuber and another produced by Corazza and Villanacci [1], [3], [4].These classifications are practical in clinical work, but allocation to specific groups may be challenging and minor histologic changes are easily missed [4]. Such small but significant changes can be caused even by relatively small amounts of gluten and are particularly important when the clinical effectiveness of pharmacological therapies is evaluated.

Also noteworthy is that only a morphologically healed mucosa, and not the disappearance of symptoms, is a prerequisite for the long-term well-being of a patient [5]. The key issue is thus whether a potential drug or vaccine is able to prevent or attenuate the gluten-induced mucosal damage [6], [7]. Optimally, histological evaluation should be made with validated and reliable readout tools [4]. Unfortunately, recent studies have shown poor reproducibility when using the results of grouped classification as the primary outcome. Moreover, evaluators have given discrepant results in borderline findings, which are diagnostically the most critical part of the deterioration [8]–[10]. An important reason for these diagnostic problems might be incorrect orientation of biopsy specimens, leading to tangential cuttings and faulty interpretations [11]–[14]. Hence, validated standard operating procedures (SOPs) are needed to ensure reliable and reproducible results.

We here evaluated the quantitative morphological (villus height-crypt depth ratio, VH:CrD) and inflammatory (density of intraepithelial lymphocytes, IEL) variables used in the assessment of different degrees of damage in small-intestinal mucosal biopsies. The aim was to provide a standardized methodology and cut-off values for significant gluten-induced changes in the small-intestinal mucosa to be employed in routine clinical practice, and in academic and pharmacological studies in celiac disease.

## Materials and Methods

### Study design and small-bowel mucosal specimens

The study was conducted in the University of Tampere and Tampere University Hospital. The material comprised altogether 93 small-intestinal mucosal specimens from 72 patients, which were obtained from the prospectively collected database and biobank maintained by our study group. Altogether 16 specimens were obtained from newly diagnosed untreated celiac disease patients, 44 from patients on a gluten-free diet, 16 from patients who underwent gluten challenge and 17 specimens from non-celiac disease controls. The mean age of the celiac patients was 57 years (range 15–81) and 67% were women. The mean age of the non-celiac controls was 50 years (range 21–73 and 59% of them were women. The small-bowel biopsies were selected (see later) to represent variable stages of mucosal injury ranging from completely normal histology to overt mucosal atrophy and crypt hyperplasia. Further, specimens with good (n = 81) or poor orientation (n = 12) were included.

The forceps biopsy specimens were formalin-fixed and embedded in paraffin and another set of biopsies from the same patient were embedded in optimal cutting temperature compound, snap-frozen and stored at −70°C until used. For morphometric analyses, paraffin-embedded standard 2-µm-thick sections were processed and stained with hematoxylin-eosin (HE) and additional sections from the same biopsy block were stained with monoclonal CD3 -specific antibody (CD3, Ab-2; Thermo Scientific, Waltham, MA). The small-intestinal mucosal VH:CrD was evaluated from at least three separate VH:CrD units by measuring villi lengths (µm) and crypt depths (µm), and the result was given as the average of the ratios. IEL densities were counted under light microscopy both in HE-stained sections [15] and in sections where T lymphocytes were stained with monoclonal CD3-specific antibodies [16]. At least 300 epithelial cells were counted in a continuous length of the epithelium, and results were expressed as number of IELs per 100 epithelial cells.

Frozen 5-µm-thick sections were processed and CD3^+^ IELs stained with monoclonal antibody Leu-4 (Becton Dickinson, San Jose, CA). The IELs were then counted with a 100× flat-field light-microscope objective throughout the surface epithelium; at least 30 fields measuring 1.6 mm in epithelial length were counted and the IEL density was expressed as cells/mm of epithelium [17].

The selected paraffin-embedded biopsy blocks comprised a wide spectrum of different degrees of mucosal injury and VH:CrD and density of CD3^+^ IELs were evaluated in parallel by two readers (JT,OK). Also, one evaluator had prior to the present evaluation studied and given results on all of these specimens (JT). The specimens were chosen by an independent selector (MM) and all measurements were made by the evaluators in blinded fashion without knowing the results obtained by the other evaluator or the clinical data of the patients. One evaluator (JT) studied and reported results on the VH:CrD measurements and all IEL countings, while the other (OK) evaluated only the VH:CrD and IELs from CD3^+^-stained paraffin specimens.

### Biopsy readout standard operating procedure

Our study group has adopted the morphometric small-intestinal mucosal biopsy measurements, as already described by Kuitunen and co-workers in 1982 [13], and subsequently formed the currently used SOP for histological readouts. SOP includes teaching technicians to obtain correctly oriented cuttings of biopsy specimens for morphometric evaluation. A crucial step in the procedure is that an accredited evaluator, besides producing acceptable interobserver and intraobserver morphometric results, be able to identify cases with an inadequate specimen and/or poor biopsy orientation, where measurements of villus-crypt units are not viable. It is essential that only biopsies in which the plane of sectioning is perpendicular to the luminal surface be considered, as judged by the fact that the crypts of Lieberkuhn are cut longitudinally and not in cross sections [11]. In a case of poor orientation resulting in tangential cuttings the evaluator asks for recuttings until reliable morphological readouts can be obtained. In IEL measurements the results are independent of biopsy orientation and recutting of specimens is rarely needed.

### Effect of tangentially cut sections on the evaluation of villus height-crypt depth ratio

A case example from routine clinics illustrating the effect of tangential sectioning and subsequent recutting on morphological biopsy readouts is presented. To highlight the importance of correct sectioning, another formalin-fixed and paraffin-embedded biopsy specimen was cut twice so that the plane of sectioning was both perpendicular and tangential to the luminal surface. These latter biopsy slices were HE-stained and grouped in Marsh-Oberhuber classes (0, 1, 2, 3a, 3b, and 3c) by five independent pathologists who were blinded to each other's results and to the double cutting of the same biopsy block.

Further, a computerized 3D model was created to evaluate the effect of different planes of sectioning on the morphological readouts of the mucosa. First, computerized blocks were made representing small-intestinal mucosa in three different stages of morphological injury and sections of these blocks then cut in such a way that the plane of sectioning was both perpendicular and tangential to the luminal surface. The computer modelling program used was Autodesk Inventor 2008 (Autodesk, San Rafael, CA).

### Statistics

Intraobserver and interobserver variations were analysed by the Bland-Altman method, linear regression analyses and intraclass correlation coefficients (ICC) [18], [19]. In the Bland-Altman method, the differences between two quantitative measurements are plotted against the averages of the two measurements, and the results reported as the mean difference between the two measurements and limits of agreement, which are defined as the mean difference plus and minus twice the standard deviation of the differences. Twice the standard deviation was used as margin of error and a change exceeding this would thus be considered clinically significant. In the Bland-Altman plot, the x axis shows the mean of the results of the two measurements and the y axis represents the absolute difference between the two measurements. When there is an increase in the variability of the differences as the magnitude of the measurements increases, differences on the y axis are recommended to be expressed as percentages of the values on the axis (i.e. proportionally to the magnitude of the measurements) [20]. Inter-method agreement was assessed with ICC. Quantitative data were expressed as number of subjects (n), mean and ranges.

### Ethics

The patient recruitment and sample collection were approved by the Ethics Committee of Tampere University Hospital and all patients gave written informed consent.

## Results

All 12 preselected biopsy specimens with tangential cuttings rendering them unacceptable for VH:CrD analyses were correctly identified by both study evaluators. The villus heights in the remaining 81 specimens with proper orientation ranged from 1 µm to 595 µm and the crypt depths from 152 µm to 535 µm. The subsequent mean value in VH:CrD was 1.39 (range 0.01–3.23). In IEL countings, five HE-stained and five CD3^+^ paraffin specimens and seven frozen specimens were excluded for technical reasons resulting from insufficient amounts of epithelial cells. The mean density of IELs in HE-stained specimens was 32 (range 8–82) per 100 epithelial cells, in paraffin-embedded CD3^+^-stained specimens 38 (range 9–103) per 100 epithelial cells and in CD3^+^-stained frozen specimens 68 (range 12–190) cells/mm.

**Figure 1:**
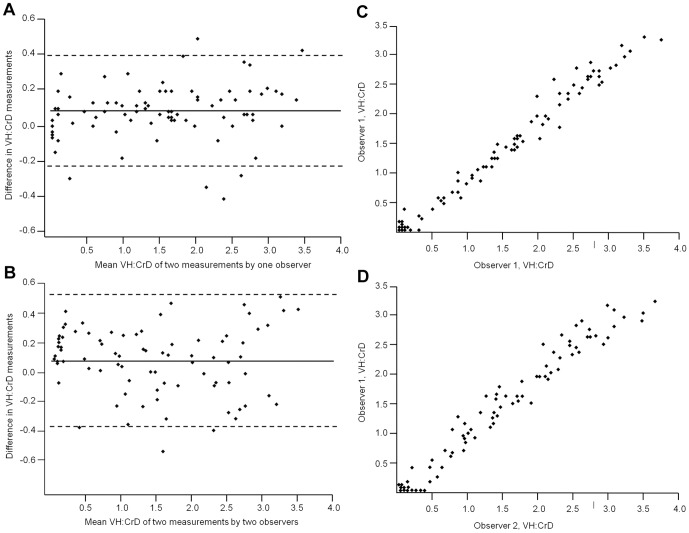
Graphical illustrations of the reliability and reproducibility of villus height crypt depth ratio. Panels A and B show the Bland-Altman plots of small-intestinal mucosal villus height crypt depth ratio and Panels C and D present the regression analyses for intraobserver and interobserver analyses, respectively. The solid lines in panels A and B indicate the mean difference between the measurements and the dashed lines correspond to the 95% limits of agreement.

The Bland-Altman plots illustrating the agreement in intraobserver and interobserver analyses in VH:CrD are shown in Figures 1A and 1B and the corresponding numerical values in Bland-Altman analyses are given in Table 1. In both intraobserver and interobserver VH:CrD analyses, the mean differences in the two measurement series compared were below 0.1, ensuring that there was no marked systematic error in the study. Twice the standard deviations, which represent the error range of the measurements, were 0.318 in intraobserver and 0.454 in interobserver analyses. The intraobserver and interobserver linear regressions are shown in Figures 1C and 1D and the ICCs in Table 1.

**Table 1:** Bland-Altman statistics with absolute values and intraclass correlation coeffients (ICC) for analysing agreement and repeatability in small-bowel mucosal villus height crypt depth ratio (VH:CrD).

	Mean difference (95% CI)	Standard deviation	Limits of agreement	ICC
**VH:CrD**				
* Intraobserver*	0.087 (0.051 to 0.121)	0.159	−0.224 to 0.398	0.983
* Interobserver*	0.070 (0.020 to 0.121)	0.227	−0.375 to 0.516	0.978

CI, confidence interval.

Bland-Altman statistics for the IEL countings are shown in Table 2. In the intraobserver analyses of IEL countings, the twice standard deviations representing the error ranges were 34.2% in CD3^+^ paraffin, 33.0% in CD3^+^ frozen and 53.2% in HE stained specimens.

**Table 2:** Bland-Altman statistics with absolute and percentage values and intraclass correlation coeffients (ICC) for analysing agreement and repeatability in the density of intraepithelial lymphocytes (IELs) of paraffin CD3^+^, frozen CD3^+^ and hematoxylin-eosin (HE) stained small-bowel mucosal biopsy specimens.

	Mean difference (95% CI)	Standard deviation	Limits of agreement	ICC
**Paraffin CD3+ IELs/100 enterocytes**				
* Intraobserver*				0.961
absolute values	1.9 (0.7 to 3.2)	5.9	−13.5 to 9.6	
percentage values	5.1 (1.4 to 8.7)	17.1	−38.6 to 28.4	
* Interobserver*				0.842
absolute values	6.9 (4.4 to 9.4)	11.6	−15.9 to 29.6	
percentage values	24.1 (18.8 to 29.4)	24.7	−24.3 to 72.5	
**Frozen CD3+ IELs/mm**				
* Intraobserver*				0.956
absolute values	2.0 (−0.3 to 4.3)	10.9	−19.4 to 23.4	
percentage values	1.0 (−2.5 to 4.5)	16.5	−31.3 to 33.3	
**HE IELs/100 enterocytes**				
* Intraobserver*				0.854
absolute values	0.7 (−2.6 to 1.3)	9.2	−17.4 to 18.7	
percentage values	8.1 (−2.5 to 13.7)	26.6	−44.0 to 60.2	

CI, confidence interval.

The ICCs presented showed excellent agreement in the CD3^+^ stainings and the agreement was good in the HE countings (Table 2). Additionally, the inter-method comparison between frozen and paraffin-embedded CD3^+^ stainings showed an ICC of 0.679 (p<0.001). These IEL densities from different fixations and stainings are mutually convertible and the following equations can be obtained from the linear regression analyses: IEL densities of paraffin-embedded CD3^+^ stainings (CD3Paraf) can be converted to the conventional IEL densities in HE-stained paraffin specimens (HEParaf) by the equation HEParaf  = 0.619 * CD3Paraf + 8.061, CD3^+^ frozen specimens (CD3Fro) to HE paraffin IEL density by the equation HEParaf  = 0.442 * CD3Fro +8.843 and IEL densities from paraffin CD3^+^ to frozen CD3^+^ staining by the equation CD3Fro  = 1.466 * CD3Paraf +10.416.

**Figure 2:**
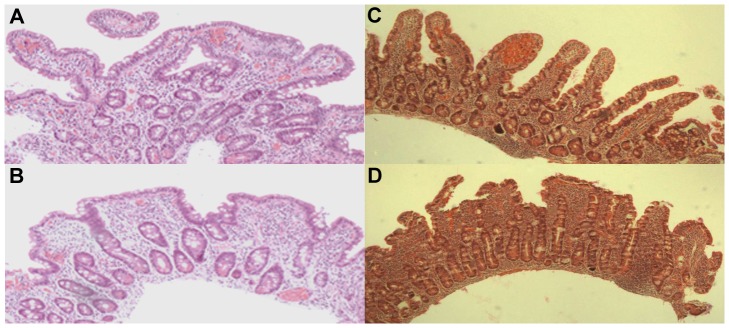
Two small-intestinal biopsy samples from routine clinics that show the importance of biopsy orientation in the interpretation of specimens. Sectionings A and C are cut tangential and B and D perpendicular to the luminal surface showing the effect of different orientation to the same small-intestinal mucosal biopsy block. The hallmark of tangential cutting is the cross-sectioning of the crypts while in correct vertical cutting the crypts are cut longitudinally. In routine clinics, the tangentially cut sectioning A was interpreted as normal, and on re-evaluation upon high clinical suspicion of celiac disease, the biopsy block was tilted and recut. The recut biopsy sample (B) reveals crypt hyperplasia and villous atrophy compatible with celiac disease. To further highlight this potential source of diagnostic error, five independent pathologists were asked to interpret another biopsy block with slices cut in different planes. All graded the tangential specimen C to be morphologically normal (Marsh 0–1) and the properly oriented specimen D to have villus atrophy and crypt hyperplasia (Marsh 3b or 3c).

**Figure 3:**
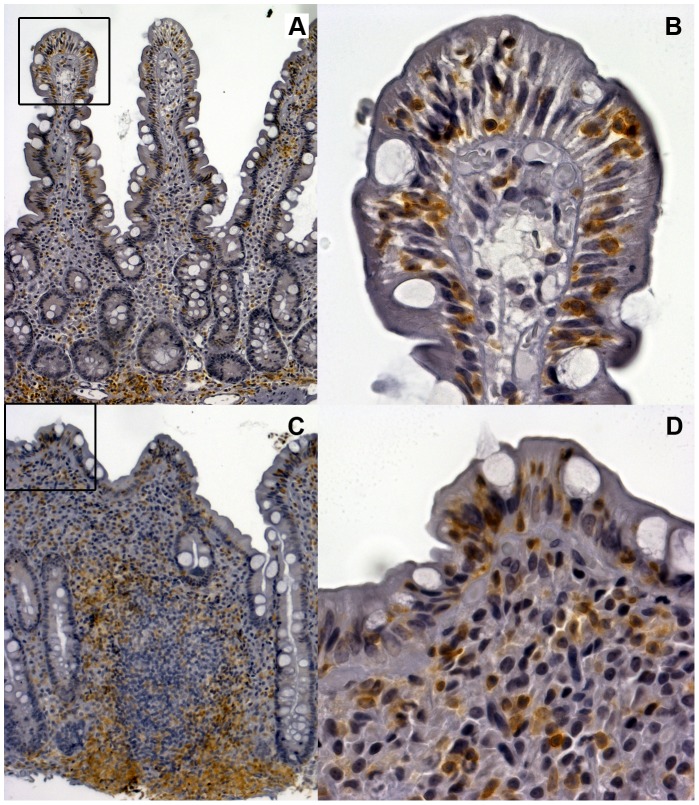
The intraepithelial lymphocyte densities are unaffected by the orientation of biopsy blocks. Panels A and C show tangential (A) and perpendicular (C) cuttings of paraffin embedded blocks stained for CD3^+^ T cells, i.e. the same biopsy block as in Figures 2C and 2D. The panels B and D present 40× magnified pictures of the mucosa from the places presented by the black rectangles in panels A and C. In these magnifications, the intraepithelial lymphocyte densities are 50 per 100 epithelial cells and 54 per 100 epithelial cells, respectively.

The effect of incorrect biopsy orientation on histological evaluation is shown in Figure 2. In routine clinics, the pathologist interpreted the specimen in Figure 2A as normal mucosa, the villous structures seen thus excluding celiac disease. However, due to a high clinical suspicion of disease, biopsy re-evaluation was asked for and recuttings performed by reason of original tangential cutting as judged by biopsy cross-sectioning of the crypts. In these reoriented specimens (Figure 2B), crypt hyperplasia with villous atrophy compatible with celiac disease was obvious. To study further this source of diagnostic error, our selected block from another patient was evaluated by five independent pathologists. All graded the tangential sectioning showing only cross-sections of crypts but tall villi as representing Marsh 0–1 (Figure 2C). When the same block had been cut perpendicular to the luminal surface, the crypts were correspondingly cut longitudinally and the same five pathologists graded the specimen as Marsh 3a–3b (Figure 2D). In contrast, no marked differences can be seen in the IEL densities in tangential and perpendicular cuttings (Figure 3).

**Figure 4:**
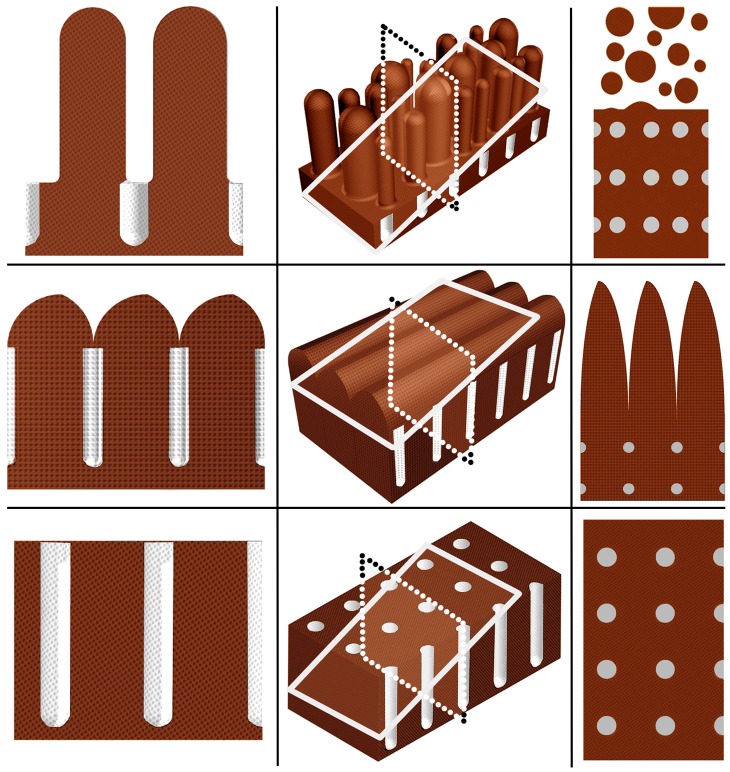
Computerized 3D-model demonstrates the effects of correct and incorrect planes of cutting on readout results. In the middle column are the biopsy blocks, in which the dashed and solid lines represent planes of sectioning. In the left column are sectionings cut perpendicular to the luminal surface and in the right tangentially cut sectionings. For example, in the middle row the computerized block shows merged and convoluted low villous ridges which in perpendicular cutting results in subtotal villous atrophy with deep crypts (left) but in tangential cutting in tall villi with only cross-sections of crypts (right).

In Figure 4, the computerized 3D-model demonstrates the effect of differing cuttings, i.e. perpendicular and tangential, in the evaluation of small-intestinal mucosal biopsy specimens. The hallmark of tangential cuttings is circular cross-sectioning of the mucosal crypts. Poor biopsy orientation results in incorrect interpretation of VH:CrD and/or incorrect Marsh class.

## Discussion

The present validation study demonstrated that quantitative variables which measure separately gluten-induced morphological (VH:CrD) and inflammatory (IEL density) changes are reliable and reproducible. Morphometric variables can be utilized for outcome measurements in both academic studies and forthcoming drug trials in celiac disease. By following our SOP, we were able to ensure strict limits of agreement in the Bland-Altman method and excellent intraobserver and interobserver ICCs in VH:CrD in biopsies with different grades of small-intestinal mucosal injury. It is obvious that the forceps biopsy specimens obtained by endoscopy are small and difficult to orientate correctly. The cornerstone of our SOP has thus been to teach the accredited evaluator when morphological variables are not amenable to measurement. In all biopsy samples the sectioning of the crypts should be inspected as a part of routine in order to avoid the reading of tangential cuttings, wherein circular cross-sections of crypts can be used as hallmark. In the present study, both accredited evaluators were able to correctly identify the specimens not suitable for subsequent morphological analyses. We believe that our methodology for biopsy orientation and measurement of morphology can be easily applied to any standard laboratory settings.

VH:CrD measurement is a sensitive tool in detecting minor changes in gluten consumption [8], [21]. We have previously used a change of 0.5 in VH:CrD as clinically relevant in assessing the effect of a gluten-free diet in celiac disease patients and also during gluten challenges [22]. The present findings would argue that this value could be reduced, since the mean difference was below 0.1 and twice the standard deviation was only 0.318 in the intraobserver Bland-Altman analysis. A cautious new cut-off value of 0.4 could thus be assigned to represent a clinically relevant difference between measurements. In the interobserver analysis again, the limit needed to obtain 95% of subjects was 0.454, indicating a slight observer-dependency in VH:CrD measurements. We infer that small but significant gluten-induced mucosal architectural changes can be reliably measured by morphometry.

The IEL densities in immunohistochemical CD3 stainings here showed better limits of agreement in the intraobserver analyses than the corresponding measurements in HE-stained specimens. In contrast, the ICC between IEL measurements of the paraffin and frozen specimens was good, and either one can be used for reliable results. It should also be noted that the frozen sections are associated with better tissue preservation than the paraffin associated specimens. However, frozen samples are more burdensome due to specific technical requirements. We have used frozen samples in academic research in order to measure, in addition to CD3^+^ cells, the αβ^+^ and γδ^+^ T cell densities [16]. This also gives an opportunity for internal staining control, as the density of all T cells (CD3^+^) should be within the magnitude of the sum of αβ^+^ and γδ^+^ T cell densities. In the present study, twice the standard deviation was in the intraobserver analysis in frozen specimens 33.0% and in paraffin specimens 34.2%. These findings agree well with previous studies in which a change of more than 30% in lymphocyte count has been considered clinically relevant [20]. The limits of agreement in the absolute IEL density values were wider in our study than those previously published, as we had substantially higher mean lymphocyte densities overall [23]. We thus provided values in percentages, as these are advocated when the variability in the measurements increases with higher IEL densities [19]. These percentage values would thus be more appropriate when assessing the effect of a gluten-free diet or novel drugs in celiac disease.

Soon after the discovery of the gluten-induced detrimental effect on the mucosal architecture in celiac disease it became clear that great care should be taken in orienting specimens in order to ensure representative vertical sections [11], [24]. One of the main reasons for missing a celiac disease diagnosis is incorrect biopsy orientation, resulting in cross-sectioning of the crypts and thus loss of evidence of crypt hyperplasia [10], [25]. In contrast, the IEL densities are mainly unaffected by the biopsy orientation as shown here in Figure 3. The importance of having complete villus-crypt units and longitudinally cut crypts is highlighted by the exemplified biopsy cuttings seen in Figures 2 and 4. Further, when what are in reality low merged and convoluted villous ridges are cut tangentially the outcome is falsely normal-looking villi, as also seen in our computerized model (Figure 4). It is also possible that a normal mucosal architecture is falsely classified as celiac disease when the cutting is almost fully tangential giving the biopsy histological appearance of a “flat” lesion. Morphologically the appearance would be similar to a tangentially cut true “flat” lesion (Figure 4). False interpretation could occur especially if no cross-sections of villi are present. These examples further demonstrate that training of evaluators to correctly assess duodenal biopsy cuttings is essential in the diagnosis of celiac disease. One possibility to obtain good quality specimens is to orientate the biopsy on acetate cellulose filter before cutting [26].

There has recently been debate as to what should be the primary outcome when evaluating the effects of new treatments in celiac disease [6], [27]. Although there is already an established mode of treatment, the gluten-free diet, there is an unmet need for novel pharmacological alternatives. Up to 60% of celiac disease patients may experience symptoms even on a strict gluten-free diet [28]. Also, mucosal healing may be incomplete with injury persisting in up to 60–80% of long-term treated patients in some series [29], [30]. Such lingering mucosal damage predisposes to celiac disease-associated complications even if symptoms disappear on a gluten-free diet [5], [31]. As a result, biopsy readouts should be favored in phase 2 clinical trials instead of self-perceived clinical outcomes. It is evident that if a drug is able to inhibit the gluten-induced mucosal injury similarly to a gluten-free diet, clinical symptoms, signs of malabsorption and serum celiac disease-specific antibodies will also disappear. In later phase 3 trials, well-validated patient-related and non-invasive biomarker outcomes can be favored as endpoints and biopsies are needed only in subgroups of patients.

### Conclusions

By following our SOP, excellent intraobserver and interobserver agreement in detecting small but significant changes in the small-intestinal mucosa can be achieved. The cut-off for clinically significant morphological duodenal mucosal healing or gluten-induced mucosal deterioration values as measured morphometrically (VH:CrD) was set as 0.4. A 30% or higher change in T-cell IEL densities, the marker of mucosal inflammation, can be regarded as clinically significant. The present results confirmed that proper orientation of biopsy specimens and recognition of incorrect tangential cuttings is crucial in ensuring reliable and reproducible histological results in celiac disease.
